# Abdominal wall hematoma after prone ventilation during postpartum VV-ECMO: a case report and systematic review

**DOI:** 10.3389/fmed.2025.1605965

**Published:** 2025-07-09

**Authors:** Guosen He, Xuemei Fan, Shuai Wang, Yu Dong

**Affiliations:** ^1^Department of Critical Care, Affiliated Hangzhou First People's Hospital, School of Medicine, Westlake University, Hangzhou, China; ^2^Department of Neurology, Affiliated Hangzhou First People's Hospital, School of Medicine, Westlake University, Hangzhou, China

**Keywords:** ARDS, prone ventilation, VV-ECMO, abdominal wall hematoma, case report

## Abstract

**Background:**

Pregnant women with ARDS are rare in themselves, and the rare complication of an abdominal wall hematoma during VV-ECMO is extremely aggressive. We report this rare case with the aim of informing clinicians to be alert to this serious complication and to share our treatment experience.

**Case presentation:**

A 42-year-old Chinese woman was infected with viral pneumonia at 26 weeks of gestation, which progressed to severe ARDS. After emergency termination of pregnancy, she was placed on VV-ECMO support and ventilated in the prone position. During this period, an unexplained drop in hemoglobin occurred, so she was withdrawn from the VV-ECMO support, and was diagnosed with an abdominal wall hematoma on CT examination, which was later cured with conservative treatment.

**Conclusion:**

Maternal prone abdominal wall hematoma found during VV-ECMO is rare and prone to adverse outcomes. We remind clinicians to be aware of this rare complication and prevent it.

## Introduction

Acute respiratory distress syndrome (ARDS), initially described as a severe complication in critically ill patients, is characterized by bilateral pulmonary infiltrates and acute hypoxemia unrelated to heart failure (1). Prone positioning ventilation is one of the few interventions proven to reduce mortality in mechanically ventilated ARDS patients (2). Although complications during prone positioning are recognized, abdominal wall hematoma is clinically rare and often presents non-specifically as an abdominal mass, acute pain, distension, or hypotension (3). Post-cesarean abdominal wall hematoma is an uncommon surgical complication; delayed diagnosis may lead to severe outcomes, including prolonged recovery, life-threatening events, and increased healthcare burden (4). We report a case of abdominal wall hematoma occurring during prone positioning ventilation under venovenous extracorporeal membrane oxygenation (VV-ECMO) support in a postpartum ARDS patient. Although the outcome was favorable, this rare complication warrants clinical vigilance.

## Patient information

**Figure 1:**
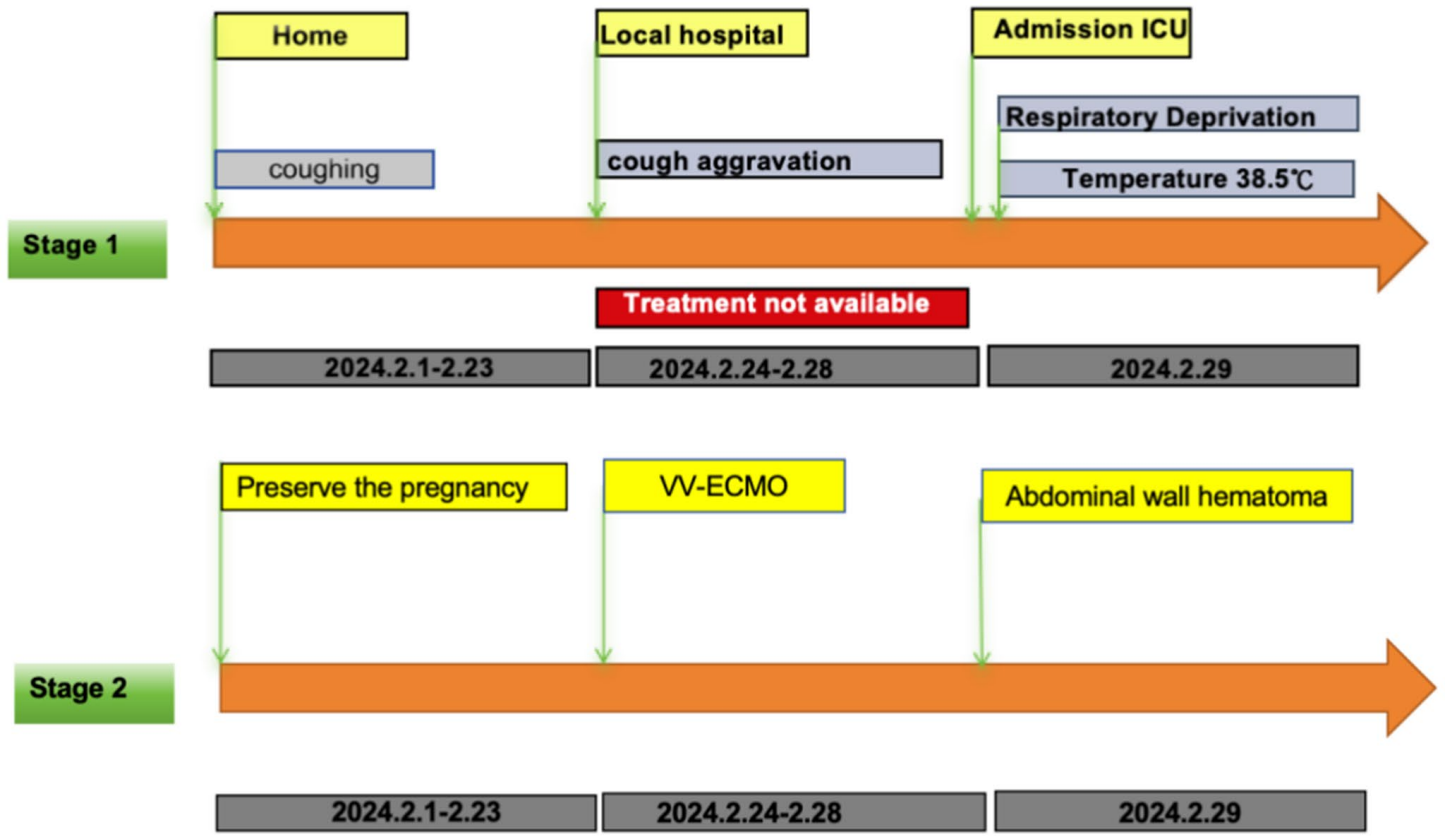
Stage 1 is the patient’s pre-admission treatment timeline; Stage 2 is the patient’s post-admission treatment timeline.

A 42-year-old woman (gravida 3, para 1, with one prior cesarean delivery) was transferred to our hospital at 26 weeks’ gestation with a 1-month history of cough and sputum. Initial tests for SARS-CoV-2, influenza A, and influenza B were negative, and community-acquired pneumonia was diagnosed. Despite 5 days of oral azithromycin (500 mg/day), symptoms worsened. On February 29, 2024, she was admitted to the high-risk maternity center and subsequently transferred to the ICU following multidisciplinary assessment (Figure 1).

### Clinical findings

**Figure 2:**
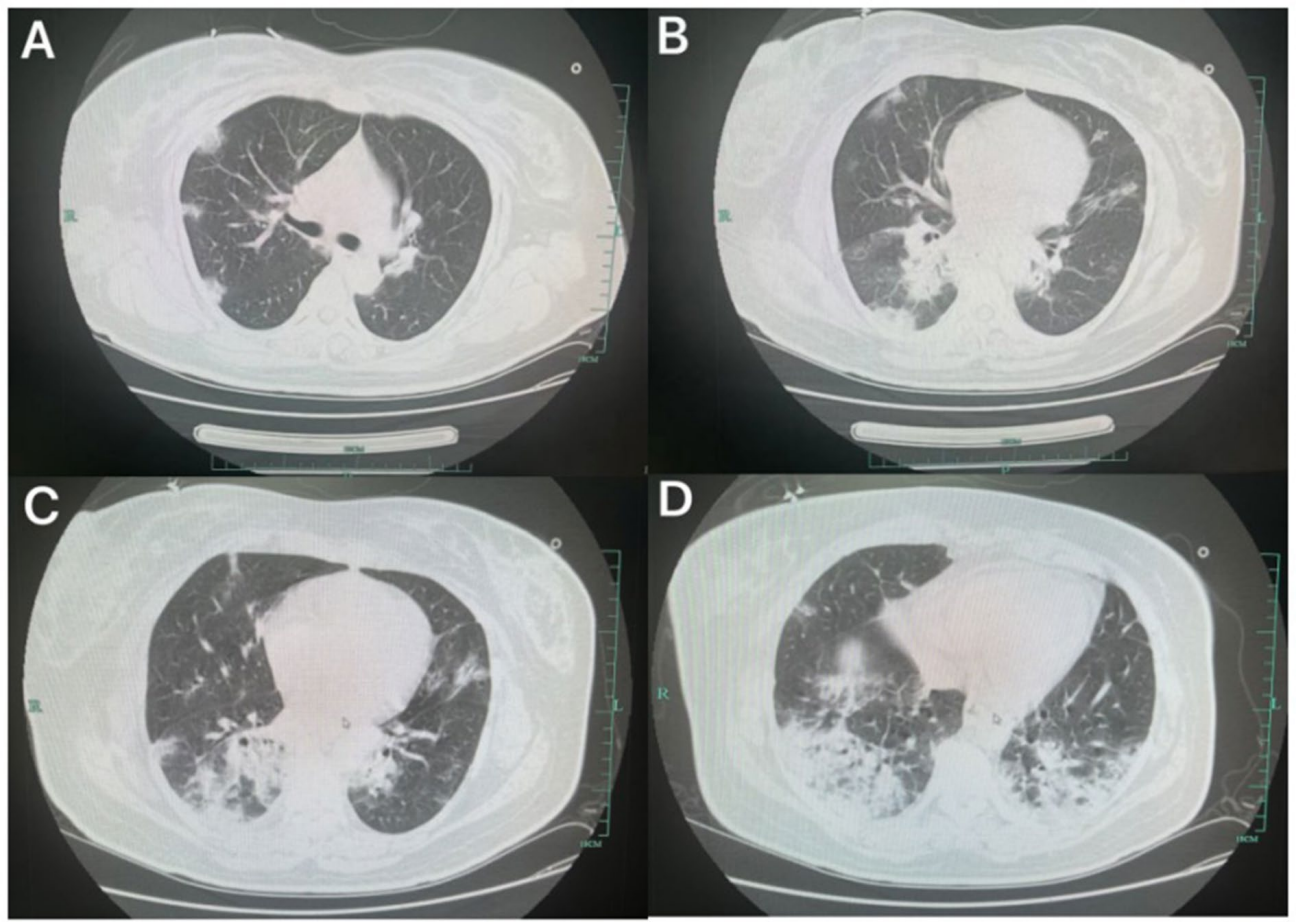
Lung CT **(A–D)** at the time of admission, showing ground-glass opacities in both lungs.

On admission, she was alert with a temperature of 38.5°C. Under high-flow oxygen therapy (FiO_₂_ 40%, 40 L/min), oxygen saturation was 91%, respiratory rate 27 breaths/min, and crackles were auscultated in the left lower lung. Laboratory results: WBC 6.7 × 10^9^/L (reference: 4–10 × 10^9^/L), neutrophils 93.1% (40–75%), lymphocytes 5.2% (20–50%), CRP 139.5 mg/L (0–8 mg/L). Lung ultrasound showed A-lines in upper fields and B-lines in diaphragmatic areas. Obstetric and cardiac ultrasounds were unremarkable. Chest CT revealed bilateral infiltrates (Figure 2).

### Diagnostic assessment

Based on clinical manifestations and etiological evidence, she was diagnosed with viral pneumonia, ARDS, and acute respiratory failure.

### Therapeutic intervention

High-flow oxygen, lateral positioning ventilation, oseltamivir (75 mg BID), and intravenous azithromycin (0.5 g QD) were initiated. Dexamethasone (6 mg Q12H × 4 doses) was administered for fetal lung maturation. By March 2, worsening hypoxemia prompted emergency cesarean delivery under general anesthesia. A 920-g female infant (Apgar 6/7) was transferred to neonatology.

Following surgery, the patient returned to the ICU with poor oxygenation, exhibiting an oxygen index (PaO_₂_/FiO_₂_ ratio) of less than 100 mmHg. Initial ventilator settings included a FiO_₂_ of 100%, positive end-expiratory pressure (PEEP) of 15 cmH_₂_O, respiratory rate of 20 breaths per minute, driving pressure of 15 cmH_₂_O, and a resulting tidal volume of 300 mL. Following discussion, prone positioning ventilation was initiated. Ventilation remained in pressure control mode during prone positioning, with settings maintained at FiO_₂_ 100%, PEEP 15 cmH_₂_O, respiratory rate 20 breaths per minute, and driving pressure 15 cmH_₂_O; the resulting tidal volume increased to 380 mL. A sustained inflation (SI) recruitment maneuver was performed, leading to improvement in respiratory mechanics and oxygenation. However, the oxygen index remained below 100 mmHg. After further discussion, VV-ECMO support was initiated on March 3. Cannulation was performed via the right femoral vein (21F drainage cannula) and right internal jugular vein (17F return cannula). Initial ECMO settings were: rotational speed 3,540 rpm, blood flow 3.67 L/min, and sweep gas flow 4 L/min.

**Figure 3:**
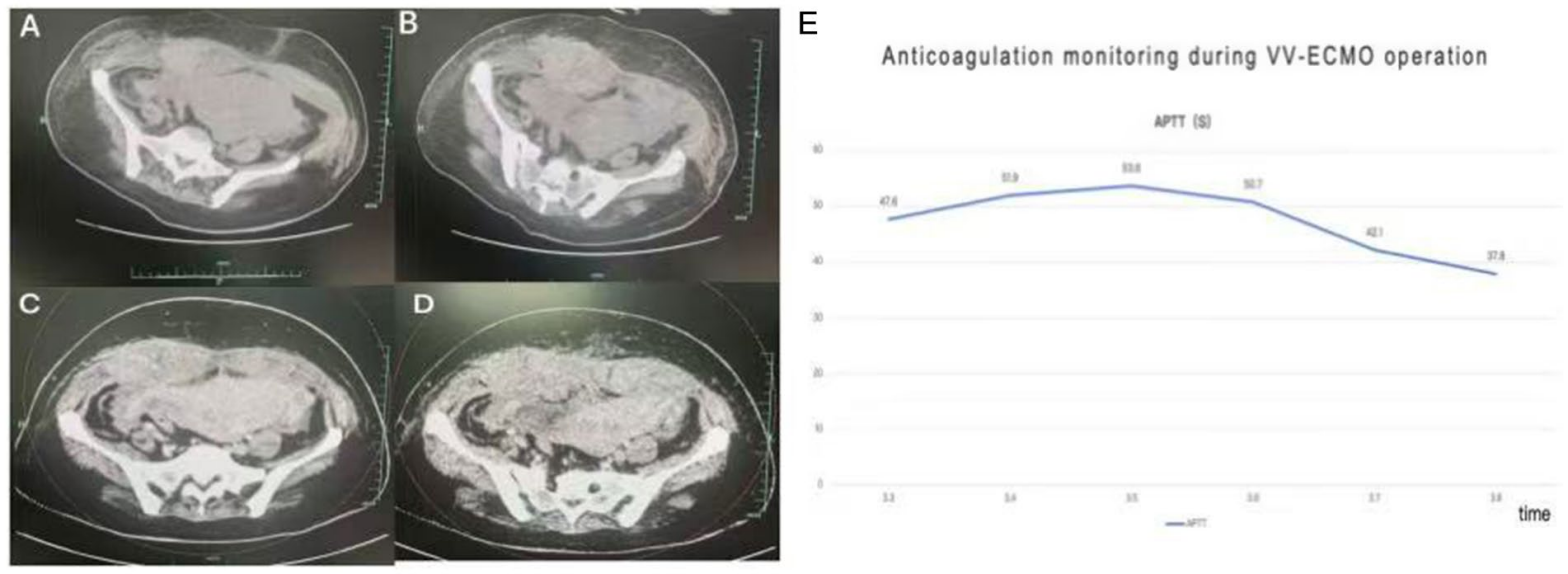
Panels **(A,B)** show a large hematoma of the abdominal wall on CT plain scan of the abdomen; panels **(C,D)** show no enhancement of the hematoma on enhanced CT; panel **(E)** shows the trend chart of the aPTT (activated partial thromboplastin time) monitoring of heparin anticoagulation during the VV-ECMO period.

In consultation with the obstetrics team, systemic heparin anticoagulation was initiated 12 h post-cesarean delivery. The starting dose was 5 units/kg/h, titrated based on activated partial thromboplastin time (aPTT) monitoring (Figure 3). Prone positioning ventilation was continued during VV-ECMO support for 12–16 h daily. During this period, a gradual decline in hemoglobin levels was observed. Repeated bedside ultrasound examinations revealed no obvious source of bleeding. On day 5 of ECMO support, a significant drop in hemoglobin occurred, accompanied by unstable ECMO flow. Anticoagulation was held, and packed red blood cells were transfused. Following assessment, ECMO support was successfully weaned and decannulated on day 6. A subsequent computed CT scan of the chest and abdomen revealed an intermuscular hematoma within the abdominal wall (Figure 3). Consultations were obtained from obstetrics and abdominal wall surgery services. Anticoagulation was withheld, and hemostasis was promoted using a double postpartum abdominal binder for compression. Hemoglobin levels subsequently stabilized. The patient was then transferred from the ICU to the abdominal wall surgery department for continued management.

### Follow-up and outcomes

**Figure 4:**
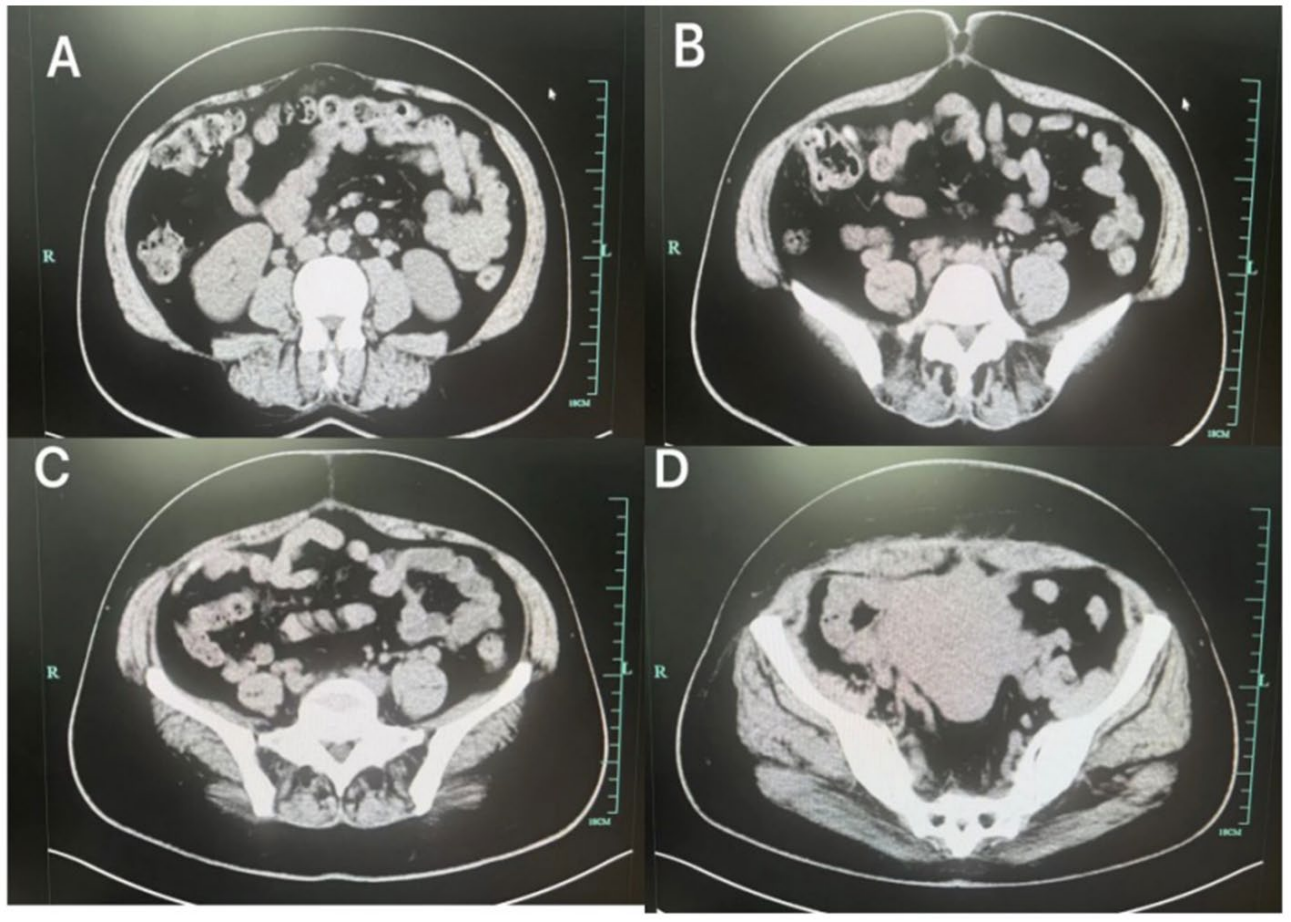
After treatment, the abdominal wall hematoma **(A–D)** was significantly reduced compared to the previous one.

Following transfer to the abdominal wall surgery department, the patient continued compression therapy with the abdominal binder and adhered well to the regimen. Repeat abdominal CT scan after 1 week demonstrated significant reduction in the size of the postpartum abdominal wall hematoma (Figure 4). Both the mother and the infant recovered fully and were discharged without long-term sequelae.

## Discussion

Abdominal wall hematoma during prone positioning under VV-ECMO is exceptionally rare. Although our patient recovered, this complication could have precipitated ECMO circuit thrombosis if it occurred earlier, increasing morbidity and costs. In the context of respiratory pandemics, this warrants heightened awareness.

Approximately 0.1–0.2% of pregnancies are complicated by respiratory failure. Physiological changes increase susceptibility to hypoxemia, which may adversely affect the fetus (5). ARDS is a leading cause of non-obstetric mortality in pregnancy (5, 6), though its incidence remains low (7). Our patient’s rapid progression to ARDS underscores the need for vigilance in similar cases.

Prone positioning, used since 1976 for ARDS (8, 9), improves oxygenation when lung-protective ventilation fails (10). Data in late pregnancy are limited to case reports (11). Our patient benefited significantly from prone positioning only after delivery, suggesting its early application in gestational ARDS may be advantageous.

Management of ARDS often requires mechanical ventilation. However, in pregnant patients receiving mechanical ventilation, excessive ventilation and alkalosis should be avoided to prevent uterine vasoconstriction, while hyperventilation and hypercapnia should be minimized to avert fetal respiratory acidosis. Additionally, maternal PaO₂ should be maintained above 70 mmHg to ensure adequate fetal oxygenation (5). Importantly, due to the rarity of ARDS in pregnancy prior to the pandemic, management strategies for respiratory compromise in this population were largely extrapolated from non-pregnant patients.

For ARDS patients managed with mechanical ventilation employing high PEEP, low tidal volume, low plateau pressure, and permissive hypercapnia, the therapeutic cornerstone remains ventilator synchronization using sedation combined with prone positioning ventilation. Prone positioning has been utilized during pregnancy and reported in case series (12–15). It improves ventilation-perfusion matching, reduces compression of the posterior and medial lungs, thereby diminishing hypoxic vasoconstriction, enhancing cardiac output, and recruiting additional alveolar units. Although randomized controlled trials are lacking, documented complications in standard ARDS patients include barotrauma, bleeding, transient hypotension, and transient hypoxemia (16), all potentially detrimental to the fetus. The need for delivery should be coordinated with neonatologists and obstetricians. Based on our prior experience and this case, timely termination of pregnancy becomes paramount when maternal condition deteriorates. Most intensivists possess limited experience managing pregnant patients with ARDS. Nevertheless, the fundamental management goals for all ARDS patients are consistent: optimizing oxygenation and maintaining perfusion and cardiac output. Crucially, potential adverse effects of therapy on the fetus must be considered to ensure the survival of both mother and fetus under the threat of hypoxemia.

VV-ECMO is reserved for refractory hypoxemia/hypercapnia (17, 18). Pre-ECMO prone positioning correlates with better outcomes (18), but delayed ECMO initiation risks irreversible lung injury. Delivery timing remains challenging. For patients >30–32 weeks with progressive respiratory failure, early delivery should be considered (19, 20). In our case, multidisciplinary debate prioritized maternal safety over fetal gestation extension. Given the gestational age of only 27 weeks, the obstetrics and neonatology teams advocated prolonging the pregnancy to enhance fetal viability. However, from the intensive care perspective, continuing the pregnancy posed extreme risks to both mother and fetus. Thus, in such scenarios, intensivists should prioritize maternal safety and advocate for timely pregnancy termination.

Cases of thoracic hemorrhage have been reported in COVID-19 (21). Management of postpartum abdominal wall hematoma lacks established guidelines. These hematomas are classified as subcutaneous, subfascial, or extraperitoneal. Subcutaneous hematomas involve superficial bleeding, presenting as wound oozing or ecchymosis, and are readily detectable. Subfascial and extraperitoneal hematomas are deeper and often identified later. Extraperitoneal hematomas, developing within loose connective tissue, can expand significantly, forming large collections. Patients typically present with low-grade fever; examination may reveal swelling, widening, and tenderness around the incision. Diagnosis is confirmed by aspiration of blood or ultrasound. Large extraperitoneal hematomas can cause marked abdominal distension, anemia disproportionate to visible blood loss, and potentially life-threatening hemorrhagic shock (22). Hematomas < 5 cm in diameter, if localized with no active bleeding, can be managed conservatively with infection prophylaxis and observation. Active bleeding, signs of infection, or anticipated low success with conservative management warrant aggressive intervention, such as endovascular embolization (23) or surgical evacuation with meticulous hemostasis and drainage if needed (24). In our patient, hematoma development during prone positioning necessitated anticoagulation hold—a precarious situation during ECMO. Fortunately, the outcome was favorable.

Prone positioning ventilation in patients with recent abdominal surgery or during pregnancy remains challenging. Some consider it a relative contraindication (25, 26). Clinicians face the core dilemma of balancing the potential benefits of prone positioning against complication risks in such ARDS patients. Strategies like reducing prone duration, alternating prone/lateral positioning, or using neuromuscular blockade to reduce intra-abdominal pressure during proning require validation through prospective studies.

The timing of heparin anticoagulation initiation in our patient (12 h post-cesarean) prompted extensive discussion. During ECMO, the balance between thrombosis and hemorrhage is a daily concern. Contact between blood and the non-biological circuit surface activates coagulation pathways, consuming both procoagulant and anticoagulant factors, thereby increasing the patient’s risk for both thrombosis and bleeding (27). Consequently, routine anticoagulation is essential for ECMO patients, with the explicit goal of preventing bleeding *in vivo* and clotting ex vivo. Due to the inherent complexity of ECMO patients, guidelines lack uniform recommendations for anticoagulation targets. The 2021 ELSO Anticoagulation Guideline (28) advocates for rational interpretation of Activated Clotting Time (ACT) values over rigid targets. Suggested targets include maintaining Activated Partial Thromboplastin Time (aPTT) at 1.5–2.5 times baseline or 60–90 s, and anti-Factor Xa levels between 0.3–0.7 IU/mL (with unchanged heparin dose if within range). Chinese expert consensus (29) recommends aPTT 40–55 s, ACT 180–220 s, and anti-Factor Xa 0.3–0.7 IU/mL for most patients. Therefore, anticoagulation requires individualized management, with clinicians adjusting targets daily based on the patient’s evolving clinical status, bleeding manifestations, and coagulation parameters.

### Take-away

We remind clinicians that prone ventilation after cesarean delivery requires attention to the serious complication of abdominal wall hematoma and suggest preventive measures for patients undergoing prone ventilation with an abdominal incision. According to our practice, the use of abdominal restraints reduces the occurrence of this complication.

## Conclusion

Maternal prone abdominal wall hematoma found during VV-ECMO is rare and prone to adverse outcomes. We remind clinicians to be aware of this rare complication and prevent it.

## Data Availability

The original contributions presented in the study are included in the article/supplementary material, further inquiries can be directed to the corresponding authors.
